# Mutation screening of the *PCDH15* gene in Spanish patients with Usher syndrome type I

**Published:** 2012-06-23

**Authors:** Teresa Jaijo, Aki Oshima, Elena Aller, Carol Carney, Shin-ichi Usami, José M. Millán, William J. Kimberling

**Affiliations:** 1 Grupo de Investigación en Enfermedades Neurosensoriales. Instituto de Investigación Sanitaria IIS- La Fe, Valencia, Spain; 2 CIBER de Enfermedades Raras (CIBERER), Valencia, Spain; 3 Department of Otorhinolaryngology, Nagano Red Cross Hospital, Nagano, Japan; 4 Genetics Center, Boys Town National Research Hospital, Omaha, NE; 5 Department of Otorhinolaryngology, Shinshu University school of Medicine, Matsumoto, Japan; 6 Unidad de Genética y Diagnóstico Prenatal, Hospital Universitario La Fe, Valencia, Spain; 7 Institute for Vision Research, Center for Deaf/Blind Studies, Department of Ophthalmology, University of Iowa Carver School of Medicine, Iowa City, IA

## Abstract

**Purpose:**

*PCDH15* codes for protocadherin-15, a cell-cell adhesion protein essential in the morphogenesis and cohesion of stereocilia bundles and in the function or preservation of photoreceptor cells. Mutations in the *PCDH15* gene are responsible for Usher syndrome type I (USH1F) and non-syndromic hearing loss (DFNB23). The purpose of this work was to perform *PCDH15* mutation screening to identify the genetic cause of the disease in a cohort of Spanish patients with Usher syndrome type I and establish phenotype-genotype correlation.

**Methods:**

Mutation analysis of *PCDH15* included additional exons recently identified and was performed by direct sequencing. The screening was performed in 19 probands with USH already screened for mutations in the most prevalent USH1 genes, myosin VIIA (*MYO7A*) and cadherin-23 (*CDH23*), and for copy number variants in *PCDH15*.

**Results:**

Seven different point mutations, five novel, were detected. Including the large *PCDH15* rearrangements previously reported in our cohort of patients, a total of seven of 19 patients (36.8%) were carriers of at least one pathogenic allele. Thirteen out of the 38 screened alleles carried pathogenic *PCDH15* variants (34.2%).

**Conclusions:**

Five out of the seven point mutations reported in the present study are novel, supporting the idea that most *PCDH15* mutations are private. Furthermore, no mutational hotspots have been identified. In most patients, detected mutations led to a truncated protein, reinforcing the hypothesis that severe mutations cause the Usher I phenotype and that missense variants are mainly responsible for non-syndromic hearing impairment.

## Introduction

Usher syndrome is an autosomal recessive disorder recognized as the most frequent cause of deaf-blindness. The frequency of Usher syndrome has been estimated to be 3.2–6.2/100,000 [1,2], in Spain 4.2/100,000 [3].

The standard clinical classification of Usher syndrome has three clinical categories, types I, II, and III [4,5]. Usher syndrome type I (USH1) is characterized by severe to profound congenital hearing impairment, vestibular dysfunction, and prepubertal onset of retinitis pigmentosa (RP); type II (USH2) displays moderate to severe hearing impairment, normal vestibular function, and teenage onset of RP. Usher syndrome type III (USH3) presents with progressive hearing loss and variable vestibular function and onset of RP.

Seven loci for USH1 have been mapped so far, and five of these genes have been identified: myosin VIIA (*MYO7A*, USH1B), Usher syndrome 1C (*USH1C*, USH1C), cadherin-23 (*CDH23*, USH1D), protocadherin-15 (*PCDH15*, USH1F), and Usher syndrome 1G (*USH1G*, USH1G) [6,7]. *MYO7A* and *CDH23* are reported to be the most prevalent genes, causing 29%–55% and 19%–35% of USH1 cases, respectively [8-12]. The *PCDH15* gene is involved in 11%–19% [9,11-13], whereas the remaining USH1 genes play a minor role in the disease, its prevalence lower than 10% [9,11,14,15].

*PCDH15* was initially described to span 980 kb of genomic DNA, and several transcripts were identified [16]. The longest transcript (isoform A) comprised 33 exons and encoded a protein of 1,955 amino acids. Protocadherin-15 was composed of an extracellular domain with a signal peptide and 11 cadherin repeats (ectodomains, EC), a transmembrane, and a cytoplasmic domain (CD1). Ahmed et al. [17,18] characterized six additional exons: exon 11a encoding a new cadherin repeat, exons 25a (non-coding) and 25b that coded for a new 5′-UTR and a signal peptide, exon 34, and exons 35 and 36 that encoded two novel and alternative cytoplasmic domains, CD2 and CD3, respectively.

Protocadherin-15 was localized in the inner ear hair cell stereocilia and in the retinal photoreceptors [13]. This protein is important in the morphogenesis and cohesion of stereocilia bundles. Protocadherin-15 and cadherin-23 proteins interact to form tip links that connect the stereocilia in the inner hair cells and are thought to gate the mechanoelectrical transduction channel [19]. In the retina, strong expression in the photoreceptors, particularly in the outer photoreceptor segments, has been observed for protocadherin-15, suggesting a role in the function or maintenance of the photoreceptor cells [13].

Mutations in *PCDH15* are responsible for USH1 and for autosomal recessive non-syndromic hearing loss (DFNB23) [13]. To date, several *PCDH15* mutation screenings have been performed, and more than 30 different point mutations have been identified as well as large rearrangements, including deletions and duplications (UMD-PCDH15 Locus Specific Database [20-22]).

In the present study, we report the results of sequence analysis of all 38 coding exons of the *PCDH15* gene in 15 probands with Usher syndrome type I and four clinically non-classified USH from the Spanish population. These patients had been previously screened for mutations in *MYO7A* and *CDH23* and for copy number variants in *PCDH15*.

## Methods

### Patients

Nineteen unrelated Spanish patients were mainly recruited from the Federación de Asociaciones de Retinosis Pigmentaria de España (FARPE) and from the ear, nose, and throat (ENT) and Ophthalmology services of Spanish hospitals.

Patients were clinically diagnosed with Usher syndrome. Fifteen were classified as USH1 based on audiological tests, vestibular function, and ophthalmologic evaluations, whereas four were non-classified because of lack of data. Audiometric examination consisted on otoscopic exploration, pure-tone audiometry, and speech audiometry. The ophthalmologic exploration included visual acuity measurements, fundus ophthalmoscopy, and visual field examination. Vestibular testing consisted of electronystagmography, gaze, and positional nystagmus. This cohort was selected because previous studies had discarded the *MYO7A* and *CDH23* genes as responsible for the disease [10,12,23]. In most cases, samples from family members were obtained.

A set of 50 healthy unrelated Spanish samples were used as controls. The ethics committees from the Boys Town National Research Hospital and the Instituto de Investigación Sanitaria IIS - La Fe approved the present study. All the procedures used conformed to the tenets of the Declaration of Helsinki. Informed consent for genetic testing was obtained from all participants after the nature and possible consequences of the study were explained.

### Mutation screening

Genomic DNA was extracted from peripheral blood collected in EDTA tubes using an automated DNA extractor (MagNA Pure compact Instrument, Roche Applied Science, GmbH). All the 38 coding exons of the *PCDH15* gene and intronic sequences flanking each exon were PCR amplified using primers listed in Appendix 1 [16,17]. Products were sequenced following the manufacturer’s instructions (Applied Biosystems, Carlsbad, CA), and the obtained sequences were compared with the corresponding reference sequences (NM_033056; EU718480 for CD2-isoform 1; EU718481 for CD2-isoform 2; and EU718482 for CD3) using the BLAST program. Whenever two putative pathologic variants were identified in a patient, segregation analysis was performed on all available family members to confirm the cosegregation with the disease and to determine parental origin.

### Computational analysis

Splice-site variants were in silico analyzed to predict their effect on the donor and acceptor splice sites. Two programs were used: the Splice Site Prediction by Neural Network (NNSPLICE 0.9) and Spliceview.

Missense changes were studied using different programs to predict whether variants are deleterious. PolyPhen classifies an amino acid substitution as probably damaging, possibly damaging, benign, or unknown, PMut provides a binary prediction of neutral or pathologic, and SIFT predicts whether a change is tolerated or deleterious.

## Results

Mutation analysis of *PCDH15* in 19 unrelated Spanish patients revealed seven different point mutations, five novel, in six patients (Table 1 and Table 2). These changes included one probably pathogenic missense variant and six pathogenic mutations: one frameshift, two splice-site mutations, and three nonsense changes.

**Table 1:** Probable pathogenic point mutations identified in Spanish Usher I patients in the *PCDH15* gene.

**Nucleotide change**	**Amino acid change**	**Exon/ intron**	**Number patients/ alleles**	**SNP**	**Domain**	**References**
**Missense**						
c.401G>A	p.R134Q	5	1/1	—	EC1	[22]
**Frameshift**						
**c.1304_1305insC**	**p.T436YfsX12**	11	1/1	—	EC4	This study
**Splice-site**						
**c.2868+5G>A**	—	21	1/1	—	EC9	This study
**c.3717+2dupT**	—	27	1/2	—	EC11	This study
**Nonsense**						
c.733C>T	p.R245X	8	1/1	rs111033260	EC2	[24]
**c.1737C>G**	**p.Y579X**	14	1/1	—	EC5	This study
**c.2782A>T**	**p.K928X**	21	1/2	—	—	This study

In bold: Variants not previously reported.

**Table 2:** Segregation analysis and clinical information of Spanish patients in whom pathogenic *PCDH15* mutations have been detected.

**Patient**	**Paternal allele**	**Maternal allele**	**Sensorineural hearing loss**	**Vestibular function**	**Age at diagnosis of RP**	**Visual field**	**Visual acuity**	**Fundus appearance**
RP-219	c.3717+2dupT	c.3717+2dupT	Congenital, profound, stable	vestibular dysfunction	2	Concentric loss <10° (17 years)	0,3 / 0,15 (17 years)	Typical RP
RP-367	c.158–52781_475–3295dup	p.R134Q	Congenital, profound, stable	vestibular dysfunction	8	Concentric loss 10° (12 years)	0,7 / 0,8 (12 years)	Typical RP + macular involvement
RP-576M	p.K928X	p.K928X	Congenital, profound, stable	ND	22	ND	0,8 / 0,7 (23 years)	Typical RP + macular involvement
RP-982	c.158–52781_475–3295dup	c.158–52781_475–3295dup	Congenital, profound, stable	vestibular dysfunction	7–8	Concentric loss. 10° (32 years)	0,6 /0,6 (32 years)	Typical RP
RP-1034	c.92–13779_157+41368del	p.R245X	Congenital, profound, stable	vestibular dysfunction	9	Concentric loss. 5° (15 years)	0,7 /0,7 (13 years)	Typical RP
RP-1286	c.1304_1305insC	+	Congenital, profound, stable	ND	9	Concentric loss (36 years)	ND	ND
RP-1323	c.2868+5G>A	p.Y579X	Congenital, profound	ND	ND	ND	ND	RP

Age at diagnosis of RP is showed in years. ND: No data.

Segregation analysis was performed in all cases where two pathologic variants were found, and cosegregation of mutations with the disease was observed in all families.

### Missense variants

One missense change, classified as probably pathogenic, was identified in this study. Variant p.R134Q (c.401G>A) located in EC1 was detected in trans in patient RP-367, carrier of a duplication of exons 4–6 in *PCDH15* (family previously reported by Aller et al. [22]). The PMut and SIFT programs predicted this variant as neutral and tolerated. However, PolyPhen classified the variant as possibly damaging.

### Frameshift mutation

One frameshift mutation was detected in the heterozygous state in patient RP-1286. This variant consists of the insertion of a cytosine between positions c.1304 and c.1305 of the *PCDH15* gene, leading to a frameshift causing a truncated protein in the EC4 domain. This insertion is produced near the end of exon 11. Nevertheless, no effect on the splicing process was predicted by the splicing analysis programs used.

### Splice site mutations

Two splicing variants were detected in our cohort of patients. c.2868+5G>A was detected in the heterozygous state in patient RP-1323, who is also a carrier of a nonsense mutation, and variant c.3717+2dupT was found homozygously in patient RP-219. Specific splicing programs showed that these variants abolished the recognition of the consensus donor splice sites.

### Nonsense mutations

Three mutations causing direct stop codons were identified. The mutation p.R245X (c.733C>T) has been widely described in previous studies in patients with USH1 [24]. This variant is predicted to truncate the protein in the EC2 domain. In our cohort of patients, the variant was detected in a compound heterozygous patient, RP-1034, with the reported deletion comprising exon 3 of the *PCDH15* gene [22].

The novel mutation p.Y579X (c.1737C>G) identified in patient RP-1323 causes a truncation in exon 14 encoding the EC5 domain. That variant was identified in our proband together with a splice-site mutation.

Finally, we detected the mutation p.K928X (c.2782A>T) in the homozygous state in patient RP-576M, who belongs to a consanguineous family. This mutation, located in exon 21, leads to the loss of the last three EC domains as well as the transmembrane and cytoplasmic domains.

In addition to the probable pathogenic mutations listed in Table 1, 38 sequence variants were identified in our cohort of patients (Appendix 2). Eleven exonic changes were detected. Two were located in exons 35 and 36: p.E1611A (c.4832A>C) in exon 35 (protocadherin-15-CD2) and p.Q1654P (c.4961A>C) in exon 36 (protocadherin-15-CD3). The remaining 27 variants were intronic. Based upon previous studies, segregation analysis, bioinformatic predictions, and allele frequencies, these 38 variants were classified as non-pathogenic.

Clinical data from patients with at least one clear pathologic mutation are shown in Table 2. In *PCDH15* patients for whom detailed clinical information was obtained, a typical Usher syndrome type I phenotype was observed. They were diagnosed with profound sensorineural hearing impairment before 1 year of age, and at least in three cases vestibular dysfunction was present. The age at diagnosis of RP occurred before 10 years, with the exception of patient RP-576M, who showed the first symptoms of the disease at the age of 22. Eye fundus revealed attenuation of vessels, waxy pallor of the optic nerve head, and bone spicules deposits (typical RP). Two patients also had macular involvement. The visual field loss was concentric, and the degree of visual acuity ranged from 0.15 to 0.8, depending on the age at ophthalmological evaluation and the degree of progression of the disease.

## Discussion

In the present report, we describe the results obtained from the mutation screening of the *PCDH15* gene in a series of 19 Spanish patients with USH in whom *MYO7A* and *CDH23* had been previously discarded as disease-causative. This study includes the analysis of the additional coding exons as well as the results of the screening of large rearrangements involving *PCDH15* previously reported [22].

Seven different point mutations were detected, two in the homozygous state. Including the large deletion and duplication detected in our patients with Multiplex Ligation-dependent Probe Amplification (MLPA) and Comparative Genomic Hybidization (CGH) array [22], seven patients were carriers of at least one pathologic variant (36.8%). Thirteen pathologic *PCDH15* alleles out of the 38 screened alleles were detected (34.2%).

All detected mutations have a clear pathogenic effect with the exception of the missense variant p.R134Q. It has been considered probable pathogenic because it was not detected in control samples and because it was found in trans together with a large *PCDH15* duplication. Mutation p.R134G was described as responsible for non-syndromic recessive deafness in two consanguineous families from Pakistan [13,17]. This may indicate an important role for the amino acid R134 in the protein.

Two missense variants, p.N174S and p.R1273S, were detected in very low frequency (see Appendix 2). The novel variant p.N174S was identified in one allele of patient RP-1321, also a carrier of variant p.Q1496H (classified as pathogenic) located in the *CDH23* gene [12]. Both genes are located in the same chromosome (*PCDH15* in 10q21.1 and *CDH23* in 10q22.3). Segregation analysis was performed in this family, and we observed that both missense variants were located in cis. These results do not support a possible digenic inheritance between *CDH23* and *PCDH15* in this family. The *PCDH15* change was not detected in control chromosomes, but bioinformatic tools do not suggest a pathologic effect of this variant. In view of these results, we considered the *PCDH15* variant p.N174S probably non-pathogenic.

Regarding variant p.R1273S, it was identified in the heterozygous state in patient RP-1387. Bioinformatic analyses showed different results, classifying this change as neutral (PMut), tolerated (SIFT), and probably damaging (PolyPhen). The change was not found in 100 control chromosomes, and the loss of a positive charge at position 1273 of the protein could affect the structure and interactions of protocadherin-15. However, a second *PCDH15* mutation was not detected. Bonnet et al. [25] reported this variant in the heterozygous state in one patient with USH1 with the second mutated allele remaining undetected. The authors classified p.R1273S as presumably pathogenic. However, we considered this change a presumed non-pathogenic variant.

Pathogenic variants were not detected in the additional *PCDH15* exons. Two missense changes were identified, p.E1611A (located in exon 35, CD2) and p.Q1654P (exon 36, CD3). Whereas the protocadherin-15-CD1 has a restricted expression profile, a wider pattern of expression was observed in protocadherin-15-CD2 and -CD3 [17]. To date, no mutation has been reported in the additional exons. This could be due to the small sample size screened for mutations or because mutations in these isoforms do not cause Usher syndrome but perhaps other disorders.

No mutational hotspots or recurrent mutations have been identified in the *PCDH15* gene, with the exception of p.R245X, a founder mutation responsible for about 50%–60% of Usher syndrome type I cases in the Ashkenazi Jewish population [24,26].

Despite the exhaustive screening of *PCDH15*, including the direct sequencing of all coding exons from known isoforms and including a search for large rearrangements, only one heterozygous mutation, c.1304_1305insC, was identified in patient RP-1286. Technical errors or hidden mutations in gene regions such as introns or regulatory sequences may account for this case. This patient may also be a carrier of a variant in *PCDH15*, and two causative mutations may be located in some other USH gene. However, we calculated that there was only a 0.0046 probability that this case is a happenstance carrier [27].

*PCDH15* mutations were detected in seven patients. All were referred to as USH1, although detailed clinical data were obtained from only six, supporting their clinical diagnosis. Mutations in *PCDH15* are responsible for Usher syndrome type I (USH1F) and DFNB23. Previous studies suggest an association of hypomorphic alleles with DFNB23 due to the conservation of residual function sufficient for normal vision but not for hearing, while severe mutations are USH1 causative [13,28]. In our cohort of patients, most of the detected pathologic variants were truncating mutations. Only in patient RP-367 was a missense variant identified in trans together with a large duplication, in whom a clear USH1 diagnosis was established. Ahmed et al. [13,17] described DFNB23 families homozygous for variant p.R134G. The phenotypic differences could be due to the second mutation (a rearrangement leading to a truncated protein in our patient with USH1), or that the missense variants had different effects on protein.

A better understanding of the consequences of *PCDH15* mutations within the different isoforms and tissues where the mutations are expressed will help shed light on which functions are conserved or damaged causing different phenotypes as USH1 or DFNB23. This knowledge will be helpful in developing therapies for these diseases.

## Appendix 1. Primers to sequence the *PCDH15* gene.

To access the data, click or select the words “Appendix 1.” This will initiate the download of a compressed (pdf) archive that contains the file. The melting temperature used to amplify these fragments was 55 °C. ^a^Primers previously described by Ahmed et al. [16]. ^b^Primers modified from Ahmed et al. [16]. ^c^Primers previously described by Ahmed et al. [17]. ^d^Primers described in the present study.

## Appendix 2. Presumed non-pathogenic alterations found in *PCDH15*.

To access the data, click or select the words “Appendix 2.” This will initiate the download of a compressed (pdf) archive that contains the file. NV: Variants not previously described. *: Nomenclature based on the protocadherin-15-CD2 (EU718480). **: Nomenclature based on the protocadherin-15-CD3 (EU718482). Variant c.986–81C>T has been previously described [11], but no SNP number has been assigned.
